# Physiological and condition-related traits in the gynogenetic-sexual *Carassius auratus* complex: different investments promoting the coexistence of two reproductive forms?

**DOI:** 10.1186/s12862-015-0438-6

**Published:** 2015-08-07

**Authors:** Andrea Šimková, Pavel Hyršl, Karel Halačka, Lukáš Vetešník

**Affiliations:** Department of Botany and Zoology, Faculty of Science, Masaryk University, Kotlářská 2, 611 37 Brno, Czech Republic; Institute of Experimental Biology, Faculty of Science, Masaryk University, Kotlářská 2, 61137 Brno, Czech Republic; Institute of Vertebrate Biology, Academy of Sciences of the Czech Republic, v.v.i., Květná 8, 603 65 Brno, Czech Republic

## Abstract

**Background:**

*Carassius auratus complex* is an extraordinary species complex including the diploid and polyploid forms exhibiting asexual and sexual reproduction modes. The coexistence of both forms in the same habitats is currently reported. The stable coexistence of asexual and sexual forms assumes some disadvantages for asexuals that balance the costs of sex. In our study, we hypothesized and tested the differences in physiological (including heamatological and immunological), growth-related, condition-related, and fitness-related traits between gynogenetic females and sexuals.

**Results:**

Our results revealed similar growth performance in gynogenetic females and sexuals measured by body size and weight, or expressed by condition factor. The energy allocation in reproduction measured by the relative size of gonads revealed no difference between gynogenetic and sexual females; in addition, both females in spawning expressed the same estradiol levels in blood plasma. We found a gender specific trade-off between investment in reproduction and immunocompetence (measured by the spleen-somatic index). Higher aerobic performance expressed by the heart index and higher oxygen-carrying capacity were found in sexual males, with increasing values before and during spawning. Our study evidenced significantly lower aerobic performance but higher oxygen-carrying capacity per erythrocyte in gynogenetic females when compared to sexuals. IgM production differed between gynogens and sexuals of *C. auratus* complex.

**Conclusions:**

Our study indicates that a similar amount of energy is invested by both gynogenetic and sexual females of *C. auratus* complex in reproductive behaviour. We suggest that lower aerobic performance in gynogens may represent their physiological disadvantage balancing the cost of sexual reproduction. A trade-off between the number of erythrocytes and the oxygen-carrying capacity per erythrocyte in sexual males and gynogenetic females may contribute to the coexistence of gynogenetic and sexual forms. In addition, the differences in specific immunity between gynogens and sexuals may also reduce the evolutionary disadvantage of sexual reproduction. In conclusion, we propose that several mechanisms contribute to the coexistence of the gynogenetic-sexual *C. auratus* complex.

## Background

The coexistence of sperm-dependent asexuals and their sexual “hosts” is a phenomenon rarely reported in vertebrates [1]. In fish, such sperm-dependent asexuals often reproduce by gynogenesis (i.e. egg development is induced by sperm of conspecific or closely-related sexual species, but there is no syngamy resulting in fully clonal progeny). To compensate the evolutionary two-fold cost of sex, the sexuals need a short-term advantage [2]. Such an advantage (i.e. some advantageous fitness components) must be frequency-dependent to stabilize the coexistence of asexual and sexual forms [3, 4].

Most models explaining the stable coexistence of asexual and sexual forms assume some disadvantages for asexuals that balance the costs of sex. According to the Red Queen hypothesis [5–7], asexual genotypes are the target of parasite adaptation, and as a result, parasites causing low fecundity and high mortality in asexual hosts may promote the coexistence of asexual and sexual forms. In relation to this hypothesis, differences in immune effectiveness between asexual and sexual forms have been predicted [8]. Potential differences in life-history traits between asexual and sexual females have been proposed on the basis of life-history regulation hypothesis [9]. Other mechanisms such as male mate choice (the behaviour regulation hypothesis [9, 10]), different feeding efficiency and competitive abilities [11, 12], and apostatic selection, i.e. the negative frequency-dependence in reproductive success or other fitness components [13], were postulated to play an important role in the coexistence of asexual and sexual forms. Finally, ecological differentiation or specialization may also mediate the coexistence of asexual and sexual forms (Frozen Niche Variation Model, [14]).

*Carassius auratus* complex [15, 16] is represented by the diploid and the polyploid forms exhibiting asexual and sexual reproduction modes. In the areas of Czech Republic, this complex recently includes the forms belonging to four mitochondrial lineages – *C. gibelio*, *C. auratus*, *C. langsdorfii* and so-called M-lineage [17]. *Carassius gibelio*, Prussian carp, is the most abundant and widespread species of *C. auratus* complex in Europe [16]. The most recent analyses of *C. auratus* complex sampled in the areas of South Moravia (Southeast part of the Czech Republic), where the presented study was conducted, indicated that 96 % of specimens morphologically identified as the representatives of *C. auratus* complex belong to the *C. gibelio* mt DNA lineage (our unpublished data). The origin of *C. gibelio* is not yet fully resolved [16]. This species is usually considered as native from central Europe to Siberia or introduced to European waters from eastern Asia [16, 18, 19]. Nevertheless, it is documented that *C. gibelio* permeated into the Czech hydrologic system by migration from the Danube River in 1975 [20]. The successful invasion of Prussian carp into novel habitats was linked with rapid reproduction due to gynogenesis (females are able to use the sperm of conspecific males or males of other cyprinid species for the activation of embryogenesis e.g. Paschos et al. [21]). Interestingly, Zou et al. [22] showed that the eggs of Prussian carp activated by sperm from common carp grew faster than those activated by sperm from conspecifics. In the experimental studies, it was showed that even triploid females are able of sexual reproduction (i.e. the eggs of triploid females may also incorporate the sperm nuclei when coexisting with diploid bisexual specimens as demonstrated by Zhou et al. [23] and the mature eggs of polyploid *C. gibelio* have three various development modes in response to sperm as demonstrated by Zhang et al. [24]). However, the low survival rates at the hatching and first-feeding larval stages of sexual triploid offspring of *C. gibelio* [23] may indicate that sexual reproduction is not a commonly adapted and successful strategy for the reproduction of triploid *C. gibelio* when coexisting with sexual diploid in the mixed populations.

In the early invasive populations of Prussian carp in the Czech Republic triploid females with gynogenetic reproduction were detected. Such a unisexual triploid character of populations had been recorded up to 1992, when the first males were recorded in the populations of Prussian carp. A few years later, *C. gibelio* had formed mixed populations composed of the sexual diploid form (with a similar proportion of females and males) and gynogenetic triploid females [19, 20, 25]. In the few last years, an increase in the numbers of sexual diploids in mixed populations of *C. gibelio* has been reported and the occurrence of solely triploid female populations seems to be very rare (Vetešník, personal observation).

A historical shift from the female gynogenetic form toward the sexual form of Prussian carp resulting in mixed populations in which both forms coexist in the same habitats may indicate that sexual diploids are currently favored over gynogens. Several studies have recently been published investigating the factors potentially contributing to the coexistence of gynogenetic and sexual forms of Prussian carp (or other representatives of the *C. auratus*-complex with dual reproduction strategies). Vetešník et al. [26], investigating the biochemical profile of blood, suggested some potential advantage for the triploid gynogenetic form (i.e. a high total protein concentration reflecting better body condition) when compared to the diploid sexual form of Prussian carp. However, they proposed that such advantages may be offset by some disadvantages such as higher concentrations of triacylglycerols and cholesterol in gynogenetic females, which may indicate a higher metabolic rate and higher energy intake when compared to sexual diploids. A parasitological survey of a mixed population of Prussian carp showed the weakened immunocompetence of triploid gynogenetic females (especially of the most common triploid major histocompatibility complex (MHC) genotype) measured by parasite load, i.e. the level of infection by gill monogenean parasites, when compared to that in diploid sexuals [27]. In addition, differential gene expression in fully-grown oocytes between gynogenetic and sexual forms of Prussian carp was recorded, suggesting different responses and behaviours of the oocytes towards sperm [28].

In the present study, we tested whether the two forms of *C. auratus* complex with different reproduction modes (i.e. gynogenetic versus sexual) should exhibit some differences in physiological, condition-related, growth-related and fitness-related traits in order to facilitate their coexistence. Such differences may reflect the advantages of one form relative to the other and may also potentially explain the historical shift from gynogenetic unisexual populations to the mixed diploid-polyploid populations.

## Methods

### Fish samples

A total of 157 individuals of *C. auratus* complex was collected by electrofishing from the River Dyje near the city of Břeclav (48° 38′ N; 16° 56 E; the Morava River basin) on 2nd November (7 diploid males, 14 diploid females and 19 triploid females; water temperature 5.4 °C), on 22nd March (5 diploid males, 14 diploid females and 20 triploid females; water temperature 7.7 °C), on 31st May (10 diploid males, 13 diploid females and 16 triploid females; water temperature 16.9 °C), and on 24th August (15 diploid males, 12 diploid females and 12 triploid females; water temperature 19.8 °C). The samples represented autumn, early spring, late spring, and summer, respectively. For our study, fish of the same age (5+ year old) were selected. Age was determined using scales following Holčík & Hensel [29].

A sample of blood sampling was collected from each individual by puncturing the caudal blood vessel using a heparinized syringe. Heparin was used as an anticoagulant (Zentiva a.s., Prague, Czech Republic) at a concentration of 50 U/ml. After sampling, the blood was centrifuged and plasma samples were stored in a freezer.

From each fish, finclip about 1 cm^2^ was taken for ploidy detection and fixed in 70 % ethanol. Before analysis this tissue was homogenized with scissors on Petri dish in 2 ml solution of CyStain DNA 1 step PARTEC and relative DNA content was estimated using Partec CCA I flow cytometer (Partec GmbH; www.sysmex-partec.com). Fresh blood of diploid *Carassius auratus* was used as reference standard.

At the end of the experiment, all individuals were euthanized by an overdose of anaesthetic (2-phenoxy-ethanol). For each individual, the standard length and total weight were measured, and the sex was determined. In addition, the somatic body weight, spleen weight, hepatopancreas weight, gonad weight, heart weight, intestine weight and length (in mm) were measured. Intestine weight was measured after removing all intestinal content. In all statistical analyses (see below) the intestine length was corrected for standard length using ratio of intestine length and standard length and intestine weight was corrected for total weight using the ratio of intestine weight and total weight. The following body indexes reflecting investments in body condition, vitality, and reproduction were calculated: condition factor, spleen-somatic index, hepato-somatic index, gonado-somatic index, and heart index. The condition factor (K) representing the relative body weight was calculated using the equation: K = constant × body weight (g)/(standard length [cm])^3^ according to Bolger and Connolly [30]. To evaluate the potential difference in growth performance between gynogens and sexuals, this index was calculated (1) using somatic weight and (2) using total body weight. The spleen–somatic index (SSI)—a measure of immunocompetence—was calculated as spleen weight (g)/total weight (g) × 100. Similarly, the relative size of the gonads (i.e. the gonado-somatic index, GSI) was calculated as GSI = gonad weight (g)/total weight (g) × 100, and the relative size of the liver (i.e. the hepato-somatic index, HSI) as HSI = hepatopancreas weight (g)/total weight (g) × 100. Finally, the heart index (HI), reflecting heart functional capacity, was calculated as heart weight (g)/total weight (g) × 100.

Animal care was in accordance with Law No. 207/2004 of the Collections of Laws of the Czech Republic on the Protection, Breeding and Use of Experimental Animals. This study was performed following the project of experiments n. 031/2011 approved by the Animal Care and Use Committee of the Faculty of Science, Masaryk University (Czech Republic).

### Haematological analyses

Immediately after sampling, erythrocyte count, haematocrit value, haemoglobin content, and leukocyte count were determined according to Svobodová et al. [31]. Erythrocyte and leukocyte counts were performed in Bürker’s haemocytometer after staining with Natt–Herrick solution. Heparinized microcapillaries (75 mm) were used to measure haematocrit and leukocrit. Blood samples were centrifuged in microcapillaries using a haematocrit centrifuge at 12.000 g for 3 min. Haemoglobin content (Hb) was analyzed photometrically (540 nm; Helios Unicam, USA) in Kampen–Zijlster transformation.

### Immunological analyses

Lysozyme concentration, complement activity, and respiratory activity were analyzed as the measures of non-specific immunity. The lysozyme concentration in skin mucus (in mg.ml^−1^) was determined by radial diffusion in agarose containing *Micrococcus luteus* (CCM 169) according to Poisot et al. [32].

Complement activity was measured according to Buchtíková et al. [33]. The total complement activity (including all activation pathways) of plasma was determined using a bioluminescent strain of *Escherichia coli* K12 (luxAmp, kindly provided by the University of Turku, Finland). The light emission measured by an LM01-T luminometer was positively correlated with the viability of *E. coli*. The relative measure of complement activity was estimated by computing the difference between the maximum time of measurement (equal to 4 h) and the time necessary to kill 50 % of *E. coli* by complement (in h).

Respiratory burst activity was measured as luminol-enhanced chemiluminescence using an LM01-T luminometer (Immunotech, Czech Republic) and opsonized Zymosan A as activator. The reaction mixture contained 50x diluted blood in Hank’s balanced salt solution, luminol (Molecular Probes, Eugene, Oregon USA, dissolved in borate buffer, pH = 9, final concentration 10^−3^ mol.l^−1^) and Zymosan A (from *Saccharomyces cerevisiae*; Sigma, USA, opsonized by incubation with serum). The final concentration of Zymosan A in the reaction mixture was 0.25 mg.ml^−1^. The maximum intensity of respiratory burst (peak in relative light units–RLU) and total intensity of respiratory burst defined as the integral of the reaction curve area (RLU*s) were included as the measures of respiratory burst. For other details, see Buchtíková et al. [33].

IgM was analyzed as a measure of specific immunity. The total IgM level was determined using precipitation with zinc sulphate (0.7 mM ZnSO_4_.7H_2_O, pH = 5.8) [34]. IgM quantification was based on the total level of proteins in the sample, determined using commercially available kit (Bio-Rad, USA) before and after precipitation. The concentration of IgM in the sample (in g/l) was calculated as the difference between total plasma proteins and proteins in the supernatant after precipitation and centrifugation.

### Steroid hormones analyses

The level of 11-ketotestosterone (11-KT) in blood plasma was analysed using the commercial competitive enzyme immunoassay (EIA) kit (Cat. No. 582751, Cayman Chemical, Estonia). Duplicates of each sample were run in two dilutions (50× and 200×) on the plate containing wells for a blank, standards and interassay variance. All plates were then analyzed using a plate reader at 412 nm (Tecan Sunrise, USA) and the concentration of 11-KT (in pg ml^−1^) was calculated according to the manufacturer’s instructions.

Plasma estradiol levels were analysed by an EIA method (Cat. No. 582251, Cayman, Estonia). Samples diluted ten times were run in triplicate and each plate contained the wells for interassay variance, standards, and a blank. The plates with samples were analysed with a plate reader at 422 nm (Tecan Sunrise, USA) and the concentration of estradiol (in pg ml^−1^) was calculated according to the manufacturer’s instructions.

The stress hormone cortisol was determined in the plasma by a solid phase enzyme-linked immunosorbent assay (Cat. No. 1887, DRG® Cortisol ELISA, Germany), based on the principle of competitive binding. Non-diluted samples were run in duplicate and each plate contained the wells for standards and a blank. The plates with samples were analysed with a plate reader at 450 nm (Tecan Sunrise, USA) and the concentration of cortisol (in ng ml^−1^) was calculated according to the manufacturer’s instructions.

### Statistical analyses

General linear models (GLM) were used to analyze the effects of fish group (i.e. this factor takes into account the effects of ploidy and sex—triploid gynogenetic females, diploid sexual females, and diploid sexual males) and season (i.e. four periods of collection spanning one year); fish body size was included in the models as a covariate. The Tukey post-hoc test was applied for multiple comparisons. Factor ANOVA was applied to analyze the effect of fish group on standard length, total weight and somatic weight. All variables were checked for normal distribution and homogeneity of variance prior to performing GLM and ANOVA analyses. Alternatively, log-transformation, root-square transformation, or arcsin-transformation were applied to fulfill the above mentioned criteria. Statistical analyses were performed in Statistica 12 for Windows, StatSoft Inc.

## Results

**Fig. 1:**
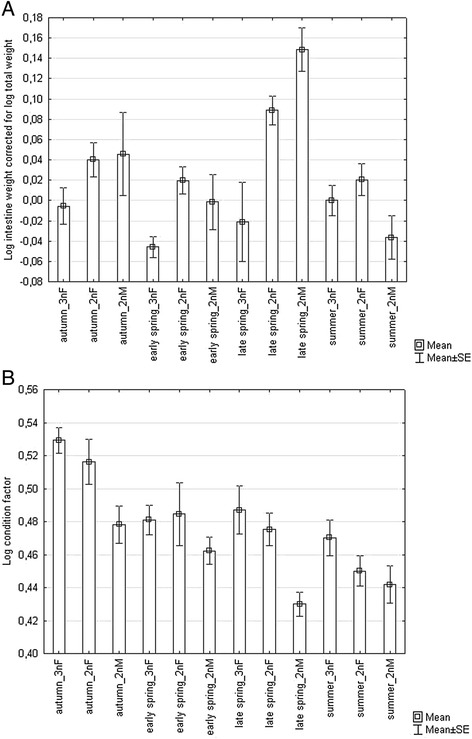
The effects of season and fish group (including 3 nF – triploid gynogenetic females, 2 nF – diploid sexual females and 2 nM – diploid sexual males) on relative weight of intestine (**a**) and condition factor (**b**)

No significant effect of fish group on body size parameters was found (p > 0.05), i.e. standard length, total body weight, and somatic body weight did not differ between fish groups. However, body size parameters differed between seasonal samples (factor ANOVA, p < 0.001), with fish exhibiting the smallest body parameter (i.e. concerning all above mentioned body parameters) in spring and the largest body parameters in summer when compared to early spring and autumn samples (Tukey post hoc test, p < 0.001). No significant effect of fish group on intestine length corrected for body length was found (p > 0.05), but a significant effect of season on the same parameter was determined (p < 0.001), with a higher value in autumn compared to other seasons (p < 0.001) and a higher value in summer compared to spring (p < 0.001). Intestine weight corrected for body weight was affected by both fish group and season (factor ANOVA, F = 6.196, p < 0.001; for fish group, F = 10.528, p < 0.001; and for season, F = 8.250, p < 0.001). The highest intestine weight was found in late spring when compared to summer and early spring (Tukey post hoc test, p < 0.001) and autumn (but with *p* = 0.084). A significantly lower intestine weight was found in triploid gynogenetic females when compared to both sexual females and males (p < 0.01). This pattern was evidenced in all samples except for summer, where both females reached a similar intestine weight (Fig. 1a). A higher condition index was recorded in autumn when compared to other seasons (p < 0.01) and it was also higher in early spring when compared to summer (*p* = 0.034). Significant effects of sampling period and fish group on condition factor were found (Table 1). Condition factor in males was lower when compared to both gynogenetic and sexual females (p < 0.001) (Fig. 1b). However, the effect of fish group disappeared when somatic body weight was used in the calculation of condition factor.

**Table 1:** The effects of fish group (including the effects of sex and ploidy associated with reproduction mode) and season on body condition and reproduction indexes

Dependent variable	Predicted variables	SS	Df	F	p	Total F	p
SSI	season	1.441	3	19.791	**<0.001**	10.458	**<0.001**
(*N* = 155)	fish group	0.666	2	13.709	**<0.001**		
	season*fish group	0.097	6	0.666	0.677		
HSI	season	9.460	3	234.060	**<0.001**	75.553	**<0.001**
(*N* = 155)	fish group	0.257	2	9.534	**<0.001**		
	season*fish group	0.110	6	1.358	0.235		
GSI	season	2472.748	3	186.352	**<0.001**	98.951	**<0.001**
(*N* = 156)	fish group	948.170	2	107.184	**<0.001**		
	season*fish group	673.676	6	25.385	**<0.001**		
HI	season	0.010	3	0.567	0.638	7.671	**<0.001**
(*N* = 155)	fish group	0.463	2	38.633	**<0.001**		
	season*fish group	0.029	6	0.807	0.566		
Condition factor	season	0.058	3	10.012	**<0.001**	5.958	**<0.001**
(*N* = 157)	fish group	0.032	2	8.327	**<0.001**		
	season*fish group	0.008	6	0.715	0.638		

The statistically significant p-values are shown in bold

**Fig. 2:**
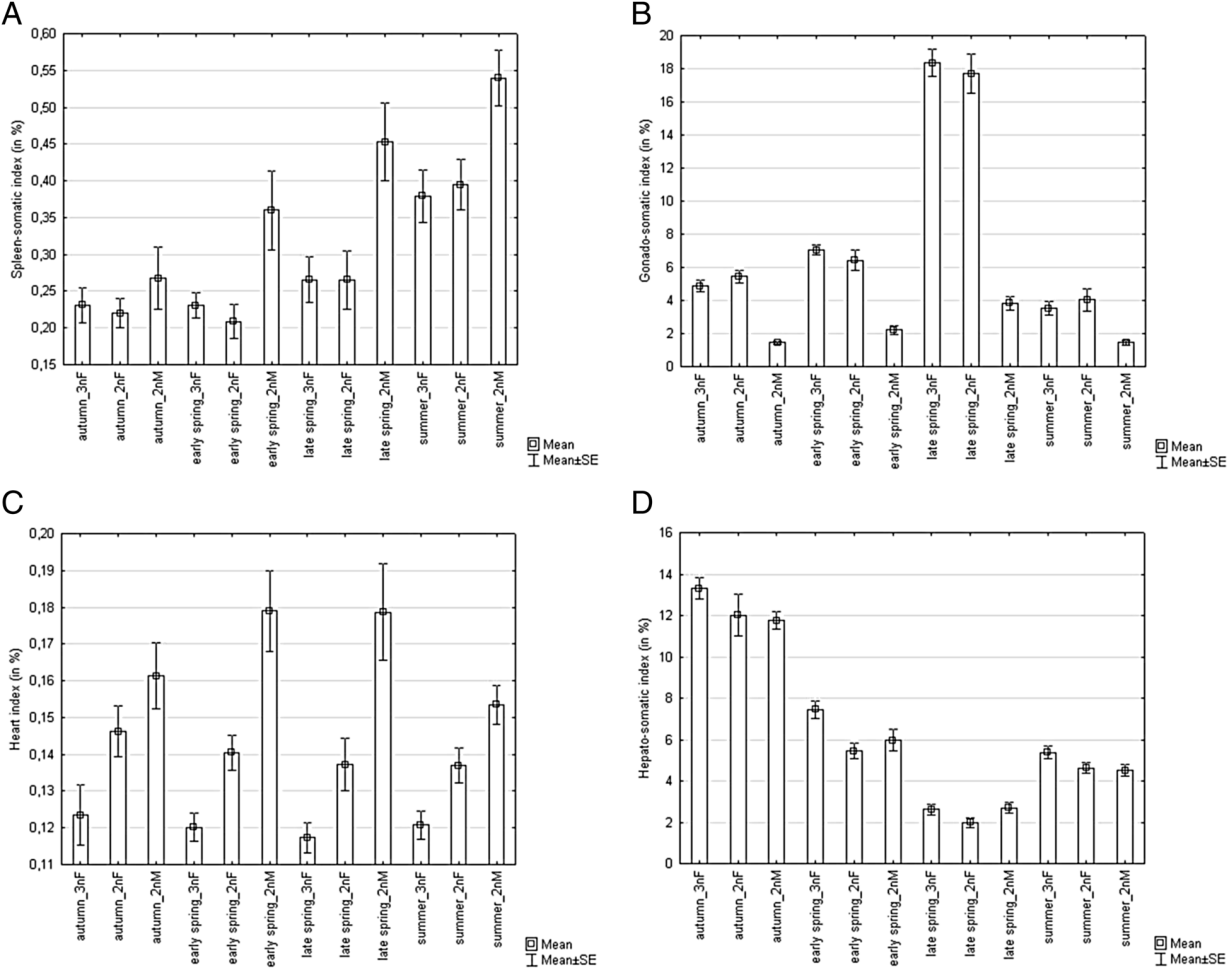
The effects of season and fish group (including 3 nF – triploid gynogenetic females, 2 nF – diploid sexual females and 2 nM – diploid sexual males) on condition-related and fitness - related traits **a** spleen-somatic index, **b** gonado-somatic index, **c** heart index, **d** hepato-somatic index

Concerning organo-somatic indexes (Table 1), all of them except for HI were affected by season. A significant effect of fish group on all indexes was found. The index of reproductive investment, GSI, was also significantly affected by the interaction of season and fish group. SSI reached its significantly lowest values in autumn and early spring; it increased in late spring (p < 0.01) and reached its highest values in summer (p < 0.001). Higher values of SSI in diploid sexual males when compared to both diploid sexual and triploid gynogenetic females (p < 0.001) were found. This pattern was recognized within each season (Fig. 2a). The significantly highest value of GSI was recorded in late spring (p < 0.001); this index then decreased in summer, but increased again from summer to autumn and from autumn to early spring (p < 0.001). The GSI of both female forms was significantly higher than GSI in males (p < 0.001), whilst no significant difference in GSI between sexual and gynogenetic females was found (p > 0.05). This pattern was well evidenced within each season (Fig. 2b). A significant effect of fish group on relative heart size (HI) was found; this index reached its highest value in 2n sexual males followed by sexual 2n females. The lowest HI values were found in triploid gynogenetic females (Fig. 2c). On the other hand, HSI (Fig. 2d) reached its highest values in autumn (p < 0.001) and decreased significantly in the following order: early spring, summer, and late spring (p < 0.001). HSI was significantly higher in triploid gynogenetic females when compared to both sexual males and females (p < 0.001).

**Fig. 3:**
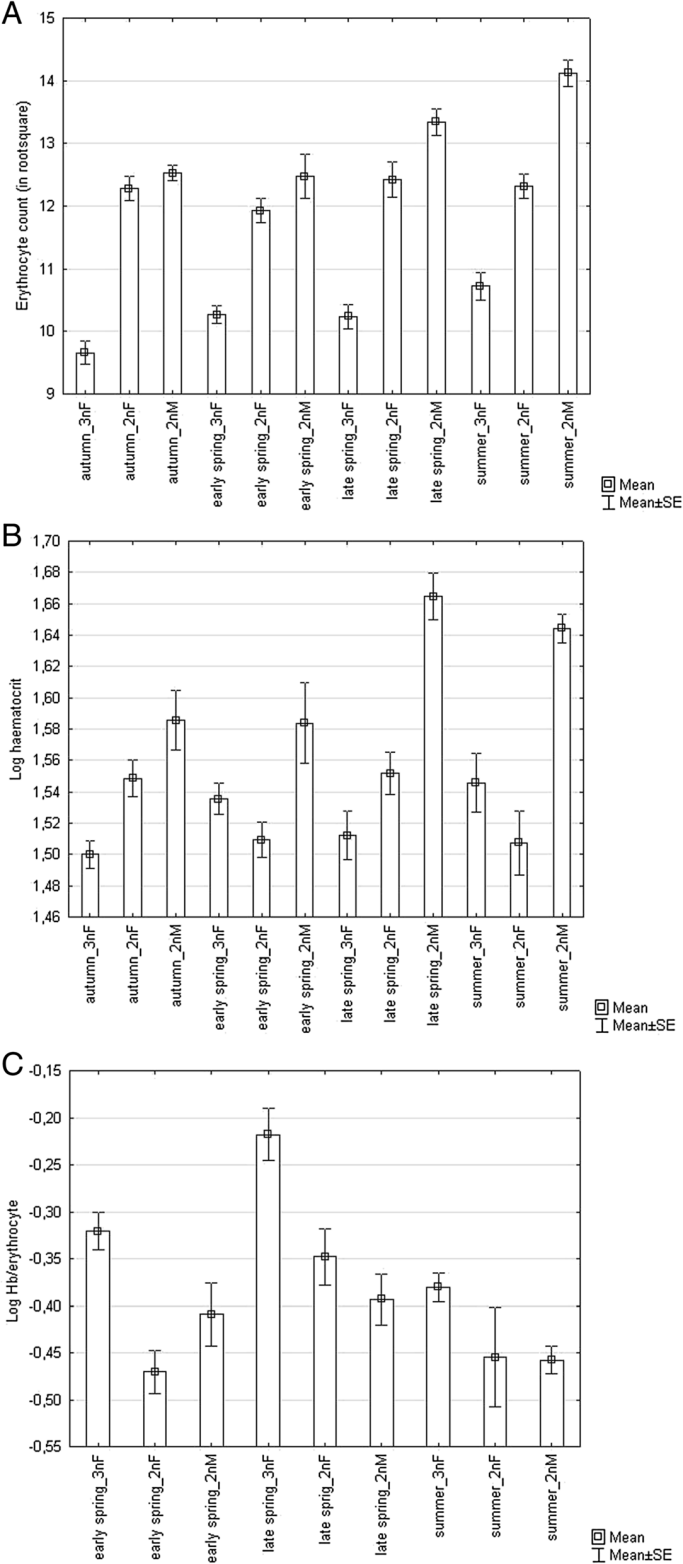
The effects of season and fish groups (including 3 nF – triploid gynogenetic females, 2 nF – diploid sexual females and 2 nM – diploid sexual males) on red blood cell parameters **a** erythrocyte count, **b** heamatocrit and **c** oxygen-carrying capacity per erythrocyte

Haematological parameters were variable throughout the different seasons. In addition, erythrocyte count, haematocrit, and Hb were significantly affected by fish group (Table 3). More specifically, all these variables had significantly higher values in sexual males when compared to gynogenetic and sexual females (p < 0.01). In addition, erythrocyte count in gynogenetic females was lower when compared to sexual females (p < 0.001) (Fig. 3a). All three variables followed the same seasonal changes, i.e. the highest values of Hb were found in late spring and the highest values of erythrocyte count and haematocrit were found in late spring and summer (p < 0.01). The seasonal variation in these parameters was the most obvious in sexual males (Fig. 3a, b). When Hb was adjusted for erythrocyte count (i.e. the oxygen-carrying capacity of individual erythrocytes) GLM revealed the significant effects of sampling period and fish group (Table 2). No significant difference was found between sexual females and males (p > 0.05), whilst gynogenetic females reached higher Hb adjusted for the number of erythrocytes (Fig. 3c). The same result was found after adjusting Hb for haematocrit values. Leukocyte count was higher in autumn when compared to other seasons (p < 0.001), whilst leukocrit reached its highest values in summer (p < 0.01).

**Table 2:** The effects of fish group (including the effects of sex and ploidy associated with reproduction mode) and season on haematological parameters

Dependent variable	Predicted variables	SS	Df	F	p	Total F	p
Erytrocyte count	body size	0.214	1	0.375	0.541	44.464	**<0.001**
(*N* = 155)	season	16.551	3	9.675	**<0.001**		
	fish group	214.126	2	187.742	**<0.001**		
	fish group*season	10.868	6	3.176	**0.006**		
Leukocyte count	body size	0.025	1	0.019	0.891	3.809	**<0.001**
(*N* = 156)	season	47.142	3	11.749	**<0.001**		
	fish group	2.774	2	1.037	0.357		
	fish group*season	5.910	6	0.736	0.621		
Haematocrit	body size	0.004	1	1.437	0.233	13.613	**<0.001**
(*N* = 155)	season	0.027	3	3.653	**0.014**		
	fish group	0.220	2	44.804	**<0.001**		
	fish group*season	0.063	6	4.259	**<0.001**		
Haemoglobin (Hb)	body size	0.001	1	0.107	0.744	5.459	**<0.001**
(*N* = 117)	season	0.152	2	6.974	**0.001**		
	fish group	0.162	2	7.424	**0.001**		
	fish group*season	0.057	4	1.297	0.276		
Hb/erythrocyte	body size	0.028	1	1.775	0.186	6.295	**<0.001**
(*N* = 116)	season	0.207	2	6.477	**0.002**		
	fish group	0.365	2	11.421	**0.001**		
	fish group*season	0.088	4	1.377	0.247		
Leukocrit	body size	0.044	1	1.646	0.202	3.762	**<0.001**
(*N* = 147)	season	0.813	3	10.217	**<0.001**		
	fish group	0.003	2	0.058	0.943		
	fish group*season	0.088	6	0.551	0.768		

The statistically significant p-values are shown in bold

**Fig. 4:**
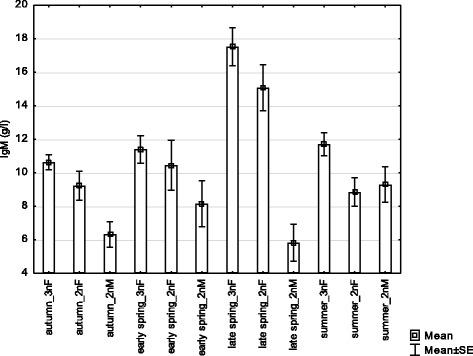
IgM concentration affected by reproductive forms (including the effect of sex) and season (3 nF – triploid gynogenetic females, 2 nF – diploid sexual females and 2 nM – diploid sexual males)

Concerning immune parameters, a significant effect of season on all immune measures was found (Table 3), with an increase in their values in spring. However, only IgM level, the single analyzed parameter of specific immunity, differed between fish groups. The IgM was significantly higher in late spring when compared to other seasons (p < 0.01), and significantly higher in gynogenetic females when compared to sexual males and females (p < 0.001). This pattern was evidenced within each period (Fig. 4). In addition, the IgM level of sexual males was also lower than that of sexual females (*p* = 0.034). Concerning measures of non-specific immunity, the highest values were found in late spring (lysozym concentration and complement activity) or in both late spring and summer (oxidative burst) (p < 0.001).

**Table 3:** The effects of fish group (including the effects of sex and ploidy associated with reproduction mode) and season on immunity parameters and steroid hormone levels

Dependent variable	Predicted variables	SS	Df	F	p	Total F	p
IgM	body size	0.032	1	0.002	0.962	8.981	**<0.001**
(*N* = 155)	season	261.658	3	6.224	**<0.001**		
	fish group	615.606	2	21.965	**<0.001**		
	fish group*season	415.222	6	4.938	**<0.001**		
Oxidative burst (integral)	body size	0.038	1	2.343	0.128	13.611	**<0.001**
(*N* = 148)	season	1.996	3	40.764	**<0.001**		
	fish group	0.012	2	0.382	0.683		
	fish group*season	0.110	6	1.120	0.354		
Oxidative burst (peak)	body size	0.272	1	4.703	**0.032**	10.272	**<0.001**
(*N* = 152)	season	4.995	3	28.742	**<0.001**		
	fish group	0.060	2	0.522	0.595		
	fish group*season	0.717	6	2.062	0.062		
Lysozyme concentration	body size	0.135	1	0.572	0.451	6.322	**<0.001**
(*N* = 132)	season	8.558	3	12.086	**<0.001**		
	fish group	0.704	2	1.492	0.229		
	fish group*season	1.955	6	1.380	0.228		
Complement activity	body size	0.010	1	1.139	0.288	14.384	**<0.001**
(*N* = 135)	season	1.287	3	48.703	**<0.001**		
	fish group	0.021	2	1.183	0.310		
	fish group*season	0.197	6	3.730	**0.002**		
Cortisol level	body size	0.189	1	2.955	0.089	5.363	**<0.001**
(*N* = 108)	season	2.801	3	14.630	**<0.001**		
	fish group	0.312	2	2.446	0.092		
	fish group*season	0.332	6	0.866	0.523		
Estradiol level	body size	0.000	1	0.000	0.984	40.453	**<0.001**
(*N* = 88)	season	10.453	3	51.065	**<0.001**		
	fish group	2.040	2	14.950	**<0.001**		
	fish group*season	6.691	6	16.342	**<0.001**		
11-ketotestosterone level	body size	0.125	1	1.131	0.295	27.994	**<0.001**
(*N* = 43)	season	5.144	3	15.477	**<0.001**		
	fish group	17.156	2	77.423	**<0.001**		

The statistically significant p-values are shown in bold

The levels of three steroid hormones—estradiol, cortisol and 11-ketotestosterone—were analyzed. Cortisol level was significantly affected by sampling period whilst the effect of fish group was not significant in the GLM model (Table 3). Estradiol level was significantly affected by both sampling period and fish group using the GLM model (Table 3). A significantly higher estradiol level was found in early spring (p < 0.001) when compared to other sampling periods. Males expressed a lower estradiol level when compared to both gynogenetic and sexual females (p < 0.001). No significant difference was found between gynogenetic and sexual females (p > 0.05). Concerning 11-ketotestosterone, the GLM model revealed the significant effects of fish group and sampling period (Table 3). Males expressed a higher 11-ketotestosterone when compared to both gynogenetic and sexual females (p < 0.001), but no significant difference was found between two groups of females. The highest values of 11-KT were found in early spring and late spring samples for males.

## Discussion

In the present study, we focussed on selected physiological, condition-related, growth-related, and fitness-related traits in the gynogenetic-sexual complex of *C. auratus*, hypothesizing some disadvantage for asexuals which should compensate the cost of sexual reproduction and facilitate the coexistence of the gynogenetic and sexual forms of *C. auratus* complex in the same habitats. In our study, we investigated specimens of the same age for both forms collected in nature.

First, we focussed on parameters of the intestine which may reflect some feeding differences between the two forms. Our analyses revealed that intestines of similar total length in gynogens and both male and female sexual forms differed in total intestine weight, i.e. significantly lower intestine weight was found in gynogenetic females when compared to the sexual forms. This finding may suggest some differences in feeding efficiency between the two reproductive forms and may indicate a disadvantage for gynogens when competing for food with sexual counterparts. However, this should be tested in the future under experimental conditions. Scharnweber et al. [12] hypothesized that the costs of sexual reproduction could be balanced if asexuals were inferior in acquiring resources. They investigated whether feeding efficiency and food competition might promote the coexistence of sexual and asexual livebearing fishes of the genus *Poecilia*. However, their study did not reveal that gynogens were less efficient foragers when compared to sexuals, and the two reproductive forms did not differ in feeding efficiency measured by gut fullness.

Next, we focussed on fitness-and condition-related traits. Mee et al. [35], predicting low asexual fitness in order to facilitate coexistence with sexual hosts, analyzed fitness-correlated traits (i.e. fecundity, egg viability, and hatching growth rate) in sperm-dependent asexual *Phoxinus eos-neogaeus* and their sexually-reproducing parental species *P. eos* and *P. neogaeus*. However, contrary to their expectation, they found a weak fitness advantage for the asexuals, which indicates that other factors should contribute to the maintenance of the coexistence of sexuals and asexuals in *Phoxinus*. In our study, we analyzed several simple measurable fitness- and condition-correlated traits. Energy allocation in the development of gonads, considered as such a fitness-related trait, was measured according to the relative size of gonads. Our study revealed that GSI did not differ between gynogenetic and sexual females of *C. gibelio*, suggesting similar allocations in gonad formation in the females of both reproductive forms. This is in line with the study by Vetešník et al. [26], who showed a high blood plasma calcium concentration (important for the development of eggs) in a spring sample for both gynogenetic and sexual females of *C. gibelio* (even the calcium in gynogenetic females was slightly higher than that in sexual females). Asexuality in fish is linked with genome polyploidization and/or hybridization, which was also the case with the *C. auratus* complex investigated here. However, the ovaries of artificially induced triploid females (in cases where polyploidization is produced for commercial purpose) are reduced in size, which results in a lower GSI and may indicate the diversion of energy from vitellogenesis to body growth [36]. Piferrer et al. [37] summarized the effects of induced triploidy on gonadal development and showed that triploids often exhibit reduced gonadal development; the presence of vitellogenic oocytes is rare in triploid females and functional gonadal sterility in females and males is observed. However, this is not the case of *C. auratus* complex when gynogenetic polyploids (triploids and rarely also tetraploids) are generated naturally. The similar values of GSI and similar body size (suggesting almost equal growth rates) most likely linked to the fertility of both sexual and gynogenetic forms were previously also documented in *Carassius auratus* by Takada and Tachihara [38]. However, other aspects of *C. gibelio* reproduction, including gonadal histology, egg viability, hatching success, and potential male mating discrimination (see below) should be examined in detail in future studies to clarify whether or not the gynogenetic form of *C. gibelio* exhibits some reproductive disadvantage promoting its coexistence with its sexual hosts.

Concerning condition-related traits, three measures of fish vigour were applied in our study—condition factor, spleen-somatic index, and hepato-somatic index. Condition factor is also considered as a measure of growth performance in fish. When comparing the growth rates of triploid and diploid fish, increased cell size does not appear to confer a growth advantage to triploids, due to the concomitant decrease in cell numbers [36]. Vetešník et al. [39], analyzing the growth of gynogenetic and sexual Prussian carp on the basis of standard length and using temporal and spatial data series collected both ten and twenty years after the introduction of Prussian carp to the Czech Republic, showed that gynogenetic triploid females had significantly higher growth rates than sexual diploids. However, condition factor calculated using somatic weight in the representatives of *C. auratus* analyzed in our study does not indicate the difference in growth performance between gynogenetic triploid females and sexual diploids. Using condition factor based on the total weight showed the difference between gynogenes and sexuals, which likely reflects the differences in gonad sizes between females and males rather than the difference in growth performance.

McLean et al. [40], in a study of coho salmon (*Oncorhynchus kisutch*), showed a reduction in condition factor caused by growth hormone in diploids but was not evident in triploids, and this hormone also caused triploids to deplete lipid energy stores more rapidly than diploids. This is in line with Vetešník et al. [26], who identified a higher metabolic rate and higher energy intake in gynogenetic females of Prussian carp when compared to sexuals. Concerning the representatives of *C. auratus* complex in our study, a higher condition was found in both female forms when compared to sexual males; the trend of a slightly higher condition was even reported for gynogenetic females when total body weight was applied in the calculation of condition factor. However, no significant difference was found when somatic weight was applied to this calculation. This finding indicates similar growth performance in both reproductive forms, but suggests the effect of ploidy and reproduction on the weight of internal organs. This is in accordance with the observation that the hepato-somatic index (considered as an indicator of fish vigour) of the representatives of *C. auratus* complex was clearly affected by the reproduction mode, with high values of the index for the gynogenetic form, supporting the hypothesis of a better condition in gynogenetic triploid females when compared to diploid sexuals, as suggested by Vetešník et al. [26].

The spleen-somatic index is often considered a simple measure of immunocompetence. Our study indicates no obvious difference in immunocompetence when comparing the two reproductive modes of *C. auratus* complex, but it does suggest gender differences in energy allocation. Thus, it seems that the high investment in gonad development in both gynogenetic and sexual females is compensated by low investment in immunocompetence (measured by spleen size), whilst the opposite trend in investments in reproduction and immunity was recorded for sexual males of *C. auratus* complex.

The heart index reflects heart functional capacity, i.e. aerobic performance, organ aerobic activity, and the animal’s ability to engage in physical activity. Generally, it has been documented that the hearts of males are larger, thinner, and have indexes of diminished performance (including lower resting heart rates) when compared to females. The differences in cardiovascular performance between genders are related to the difference in endocrine systems, i.e. the production of steroid hormones. Concerning fish, a larger heart was evidenced for mature males of migratory salmonid species investigated in the spawning period (e.g. Franklin and Davie [41]; Armstrong and West [42]; Altimiras et al. [43]; Clark et al. [44]) indicated the increased functional demands placed on the hearts of males during spawning [45]. During this period, the elevated levels of steroid hormones in salmonid males (testosterone, 11-ketotestosterone) induce hearth growth [46, 47]. On the other hand, salmonid females approaching their spawning grounds had higher heart rates and higher levels of cortisol and estradiol when compared to males [48]. Clark et al. [44] hypothesized that the increase in heart volume in salmonid males may be beneficial for increasing oxygen transport capacity during reproduction. However, they found that even if the males of sockeye salmon (*Oncorhynchus nerka*) consume significantly more oxygen than females, the females exhibited higher blood oxygen-carrying capacity measured by Hb concentration. Our data support the fact of larger heart mass in fish males when compared to females, but also show high Hb concentration in males of *C. auratus* complex, which may suggest their higher oxygen-carrying capacity. In addition, the highest heart mass and the highest Hb in males collected in spring were linked to the highest levels of 11-ketotestosterone, supporting the idea of androgen-induced heart growth with increasing aerobic performance.

To our knowledge, cardiovascular systems in diploid-polyploid complexes of fish species have not yet been analyzed. The polyploidization of the myocardium in birds and mammals is correlated with a reduction in cardiac aerobic performance expressed by heart index (for birds, see Anatskaya and Vinogradov, [49]; for mammals, see Anatskaya and Vinogradov, [50]). Genome polyploidization seems to be linked to a severe decline in aerobic respiration and to the stimulation of sugar and fatty acid metabolism, and promotes cell survival and tissue regeneration under stressful conditions in mammals, as demonstrated by analyses of the ploidy-associated changes in the expressions of non-tissue specific genes in heart and liver of human and mouse [51]. The low heart index in the triploid gynogenetic females of *C. auratus* complex may indicate low functional capacity (probably low aerobic performance). Even if the high Hb concentration in sexual males suggests their higher total oxygen-carrying capacity when compared to females (including both forms—sexual and gynogenetic), our results also demonstrate the higher oxygen-carrying capacity of individual erythrocytes of gynogenetic females when compared to sexuals. Thus, we propose that a trade-off between the high number of erythrocytes with lower oxygen-carrying capacity per erythrocyte in sexual males and the low number of erythrocytes with high oxygen-carrying capacity per erythrocyte in gynogenetic females may significantly contribute to the coexistence of both forms. Concurrently, both the increase in erythrocyte count and total oxygen-carrying capacity in sexual males and the increase in oxygen-carrying capacity per individual erythorocyte in gynogenetic females were reported in spring, i.e. during the spawning period. This phenomenon of different oxygen-carrying capacities in gynogenetic and sexual forms of *C. auratus* complex did not vary seasonally. However, morphological and functional analyses (i.e. of the cardiac energy metabolism) of cardiovascular systems as well as analyses of oxygen consumption rate may represent helpful tools for understanding the cardiovascular aspects of the evolution of physiology in the two coexisting reproductive forms of this unique fish species.

The role of immunity in the dynamics of the asexual-sexual complex was highlighted by Hakoyama et al. [52], as differences in immunity between gynogens and sexuals may reduce the evolutionary two-fold cost of sex. Whilst non-specific immunity is independent of the relative frequency of the two forms in the gynogenetic-sexual complex, differences in specific immunity may cause negative density-dependent regulation in this complex. Using parasite infection level as a measure of non-specific immunity, Hakoyama et al. [52] found the gynogenetic form to be more susceptible to parasite disease. However, Šimková et al. [27] focused on the MHC genotyping of gynogenetic and sexual specimens from the selected mixed population. They showed that infection by highly specific gill ectoparasites (*Dactylogyrus*) is higher in the most common MHC genotypes of gynogens when compared to rare MHC genotypes of gynogens or highly variable MHC genotypes of sexuals, suggesting a co-evolutionary arms-race between host immunity and parasite virulence. Our study did not reveal differences in non-specific immunity between gynogenetic and sexual forms of *C. auratus* complex measured either by respiratory burst (reflecting phagocyte activity), complement activity, or lysozym activity. We showed for the first time the clear difference in IgM production between gynogenetic and sexual forms, and this pattern was evidenced in different sampling periods throughout the year. In addition, we identified a difference in IgM production between females (both sexual and gynogenetic) and males, probably indicating the immunosuppressive roles of 11-ketotestosterone in males of *C. auratus* complex, which was especially evidenced in the spawning period. Such gender differences in IgM level were recently demonstrated in a hybridizing system of sexual Prussian carp and common carp (*Cyprinus carpio*), probably reflecting the different costs of reproductive investment in males and females [53].

The sex steroid hormones were previously suggested to play an important role in species recognition in the complex of gynogenetic and sexual fish (e.g. Gabor et al. [54]). Gynogenetic Amazon molly (*Poecilia formosa*) arose via the hybridization of female Atlantic molly (*P. mexicana*) and male sailfin molly (*P. latipinna*), this gynogenetic species requiring the sperm of their parental species. When living in sympatry with gynogenetic Amazon molly, the males of sailfin and Atlantic mollies showed a mating preference for conspecific females [55–57]. However, Gabor et al. [54] showed that males of Atlantic molly did not discriminate against gynogenetic Amazon molly as strongly as males of sailfin molly. They also showed that the levels of premating and postmating estradiol and 11-ketotestosterone are important for mating behaviour. Thus, we focused on the levels of three steroid hormones-11-ketotestosterone, estradiol, and cortisol. Whilst 11-ketotestosterone and estradiol regulate reproductive behaviour in fish, cortisol is considered as an indicator of stress (e.g. Eslamloo et al. [58]) affecting disease resistance (e.g. Fast et al. [59]; Tort [60]). Our study showed a high level of 11-ketotestosterone in sexual males of the representatives belonging to *C. auratus* complex in early and late spring, and a high level of estradiol in both gynogenetic and sexual females of *C. auratus* complex in early spring. However, cortisol level was not different in gynogenetic and sexual reproductive forms. This hormone increased during spawning (in late spring sample) for gynogens and both sexual females and males. The females of both gynogenetic and sexual forms collected during the spawning period expressed the same estradiol levels in blood plasma, probably indicating that the same amount of energy is devoted to reproductive behaviour in the females of both forms. However, only further experimental studies can clarify whether males of *C. auratus* complex discriminate between gynogenetic and sexual females in order to conserve the energy associated with sperm production [61] and, thus, whether mating choice is a factor facilitating the coexistence of gynogenetic and sexual forms belonging to *C. auratus* complex.

## Conclusions

Our study provides the first investigation of physiological and immune parameters in gynogenetic-sexual *C. auratus* complex. In addition to the limited ability of the gynogenetic form to escape parasitism as a potential mechanism promoting the coexistence of gynogenetic-sexual *C. auratus* complex [27], we hypothesized that the different investments in condition-, growth- and fitness-related traits may represent another mechanism contributing to the coexistence of gynogenetic and sexual forms of this complex. However, both gynogenetic and sexual forms of *C. auratus* complex showed comparable growth performance. Even if our study seems to indicate that similar amount of energy is devoted to the reproductive investment in gynogenetic and sexual females suggesting no reproductive disadvantage for gynogenetic form, we suggest that lower aerobic performance in gynogens (this may be amplified in eutrophic habitats with low ogygen level) may represent their physiological disadvantage balancing the cost of sexual reproduction. The different investments on the basis of a trade-off between the number of erythrocytes and the oxygen-carrying capacity per erythrocyte in sexual males and gynogenetic females may also facilitate the coexistence of gynogenetic and sexual forms. In addition, the differences in specific immunity between gynogens and sexuals may also compensate the evolutionary disadvantage of sexual reproduction. However, other mechanisms such are feeding competition, mating choice or different metabolic costs should be investigated in the future as other potential mechanisms contributing to the coexistence of this unique system. We highlight that this study may further help for the understanding invasion success of the species belonging to *C. auratus* complex in the recently invaded areas.
